# Duplicability of self-interacting human genes

**DOI:** 10.1186/1471-2148-10-160

**Published:** 2010-05-28

**Authors:** Åsa Pérez-Bercoff, Takashi Makino, Aoife McLysaght

**Affiliations:** 1 Smurfit Institute of Genetics, University of Dublin, Trinity College, Dublin 2, Ireland

## Abstract

**Background:**

There is increasing interest in the evolution of protein-protein interactions because this should ultimately be informative of the patterns of evolution of new protein functions within the cell. One model proposes that the evolution of new protein-protein interactions and protein complexes proceeds through the duplication of self-interacting genes. This model is supported by data from yeast. We examined the relationship between gene duplication and self-interaction in the human genome.

**Results:**

We investigated the patterns of self-interaction and duplication among 34808 interactions encoded by 8881 human genes, and show that self-interacting proteins are encoded by genes with higher duplicability than genes whose proteins lack this type of interaction. We show that this result is robust against the system used to define duplicate genes. Finally we compared the presence of self-interactions amongst proteins whose genes have duplicated either through whole-genome duplication (WGD) or small-scale duplication (SSD), and show that the former tend to have more interactions in general. After controlling for age differences between the two sets of duplicates this result can be explained by the time since the gene duplication.

**Conclusions:**

Genes encoding self-interacting proteins tend to have higher duplicability than proteins lacking self-interactions. Moreover these duplicate genes have more often arisen through whole-genome rather than small-scale duplication. Finally, self-interacting WGD genes tend to have more interaction partners in general in the PIN, which can be explained by their overall greater age. This work adds to our growing knowledge of the importance of contextual factors in gene duplicability.

## Background

Proteins have an impact on the cell through interactions with other components of the system. One type of interaction, Protein-Protein Interaction (PPI), has received much attention in the literature because of the possibilities of genome-wide surveys, such as yeast two-hybrid screens, and the tractability of analysis. In particular, the evolution of PPIs and how this relates to other aspects of molecular evolution is very interesting.

One special category of PPI is the interaction between identical copies of a protein produced from the same gene (self-interaction) forming homomers. These comprise a significant fraction of the protein interaction network (PIN) due to genetic factors: the interacting partners are translated from the same mRNA and so are *ipso facto *co-regulated and co-localized in the cell; as well as biochemical factors: identical proteins are expected to have high affinity for each other [1].

Gene duplication can act to shape the protein interaction network because although an identical protein copy produced from a duplicate gene will perform the same interactions as the original, over time the protein sequences and the interactions they participate in will diverge [2]. In particular, the ancestral number of interactions may influence the dynamics of gain and loss of protein interactions after gene duplication [3]. It has previously been shown that gene duplicability can be influenced by factors such as dosage-balance constraints [4], connectivity in interaction networks [5] and function [6]. The duplicability of genes also differs between small-scale (SSD) and whole genome duplication (WGD) [7,8].

Several recent studies have examined the duplication of genes whose protein product can interact with a copy of itself (for simplicity we call these "self-interacting genes") [2,9-11]. Pereira-Leal and colleagues investigated the evolutionary origins of protein complexes and concluded that they evolve through duplication of homodimers, that is through duplication of genes coding for self-interacting proteins [10]. They showed that protein interactions amongst paralogous proteins occur more frequently than can be expected purely by chance in yeast, worm and fly. They also show that protein-protein interactions between homodimers and paralogous dimers (interactions between paralogous proteins) in these species are not independent, and conclude that the latter dimer type evolved from the former, something that had been suggested previously [12]. They argue that gene duplication and divergence are important forces driving the expansion of the eukaryotic proteome, and that multiple copies of identical subunits are an economical way of forming larger functional structures. Another study, which modelled the yeast protein interaction network before and after WGD found evidence for greater retention of self-interacting genes in duplicate [11], and so is consistent with this hypothesis.

With a few exceptions (e.g., ref [9]) most previous studies have used the yeast strain *S. cerevisiae *as a model organism. This is convenient, as thorough, full-scale proteomic interaction studies have been conducted in yeast using affinity purification and mass spectronomy (AP-MS) to gain knowledge of protein complexes [13]. Moreover there is complete genome sequence of several other yeast species for evolutionary comparisons, and extensive protein interaction data between protein pairs are available in the Database of Interacting Proteins (DIP) [14].

In this study we investigate the duplicability of self-interacting genes in human. We also examine the impact of the extent of the duplication event by comparing SSD duplicate genes with WGD duplicate genes. Our results support the model of preferential retention of duplicated self-interacting genes. This result is robust against the method used to define duplicability. We also show a greater enrichment of self-interacting genes among WGD duplicates than SSD duplicates and relate this to an overall higher connectivity of WGD genes in the protein-interaction network.

## Results and Discussion

### Higher duplicability of self-interacting genes

**Figure 1:**
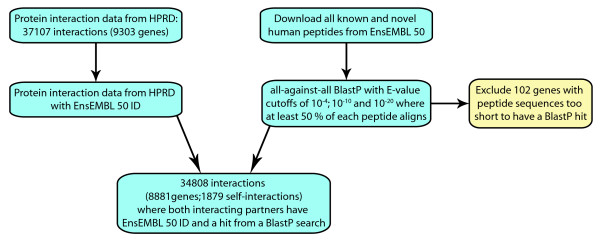
**Data collection**. Flow chart illustrating how the human interaction data were collected from HPRD release 7, and subsequently matched with blastable Ensembl Core release 50 identifiers in order to extract the final 8881 genes involved in 34808 protein-protein interactions.

We examined 34808 interactions between products of 8881 genes, 1879 of which are self-interacting (Figure 1). The protein interactions follow a power-law distribution, a typical feature of biological networks [15,16]. Singletons were defined as human genes with no BLASTP hit in the human genome (other than self-hits) at an E-value threshold of 0.1. Duplicate genes had a non-self BLASTP hit in the human genome with an E-value less than or equal to 1 × 10^-20^. Genes with hits with intermediate E-values were excluded as ambiguous. Consistent with previous studies [9,10] we found that duplicated genes are enriched for self-interactions (χ^2 ^= 45.02, p = 1.96 × 10^-11^) and this result does not depend on the E-value used to define duplicate genes (see Additional file 1).

Since homomers can give rise to heteromers through the duplication of self-interacting proteins one possible outcome of the duplication of self-interacting genes is that the self-interaction is lost as the interaction between paralogs is favoured, perhaps because it permits greater evolutionary novelty. If this is the case it may reduce the apparent duplicability of self-interacting genes when duplications and interactions are measured in the same organism [17,18]. Similarly, interactions may be gained after gene duplication. In order to exclude the possibility that the gene duplication interferes with the interaction status of the protein products we measured duplicability in a sister lineage.

**Figure 2:**
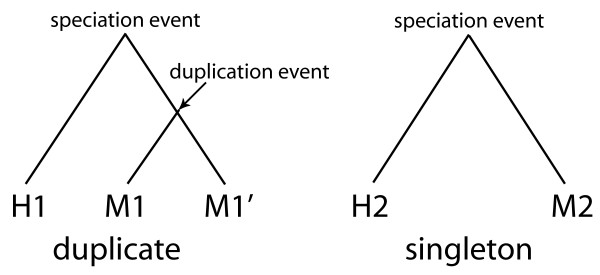
**Definition of mouse-specific duplicate genes in human**. Human genes (H) were classified as singletons if they had a one to one orthologous relationship with mouse (M), and as mouse-specific duplicate genes if the relationship with mouse was one to many *i.e*. if one human gene had at least two orthologous genes in the mouse lineage.

We searched for mouse orthologs of the human genes using Ensembl Compara and defined a singleton gene as a human gene that had a one to one ortholog in mouse, while a duplicate gene was a human gene with at least two co-orthologous genes in mouse (i.e., mouse lineage-specific duplication; Figure 2). Thus only human genes that have not experienced a recent human lineage-specific duplication (within the last 90-100 myr) are considered, and duplicability is assessed by the status in the mouse genome (Table 1). For our analysis we could only use genes with available interaction information, however the proportion of duplicated genes (mouse-specific duplication) in this reduced dataset (2.28%) is comparable to the proportion for the whole dataset (2.83%) so we infer that no bias is introduced. We find that orthologs of human self-interacting genes have greater duplicability in the mouse lineage (χ^2 ^= 3.96, df = 1, p = 0.047). The fact that the duplicated genes in this dataset are recently duplicated (since the mouse-human divergence) indicates that this is an ongoing phenomenon.

**Table 1:** Proportion of self-interacting singletons and duplicates.

Classification system		self-interacting	Total
Human BLASTP	Singletons	433 (16%)	2595
	Duplicates	1285 (23%)	5531
Mouse duplicability	1:1 orthologs	1682 (21%)	7968
	1:many orthologs	51 (27%)	186

### WGD genes are enriched for self-interactions by comparison with SSD genes

Previous studies have shown different properties for genes duplicated by SSD or WGD [19,20]. In particular, dosage-balance constraints result in different duplication outcomes under WGD (biased retention) and SSD (lower duplicability). To investigate whether the mechanism of duplication influences the retention of self-interacting genes we compared genes duplicated by the two mechanisms. It has been observed in yeast that genes that form heteromers (complexes of proteins encoded by different genes) have fewer paralogs than other genes, since the integrity of the complex depends on duplication of all, or none, of the genes in the complex [4]. In the human data, 25% of genes duplicated by WGD are self-interacting compared to only 21% of SSD-duplicated genes, a significant difference (χ^2 ^= 10.67, df = 1, p = 0.0011; Table 2). Because self-interaction does not depend on other genes this observation is not immediately reconcilable with between-gene dosage-balance explanations for WGD gene retention. However, it was previously noted that self-interacting proteins tend to have more interacting partners (higher connectivity) than non-self-interacting genes [9] and we confirm this for our dataset. Thus the higher fraction of self-interacting WGD genes may be due to an indirect effect of a greater number of interactions (and thus greater tendency for dosage-balance constraints on these genes).

**Table 2:** Proportion of self-interacting duplicate genes generated by different mechanisms.

Duplication Mechanism	self-interacting	Total
WGD	717 (25%)	2877
SSD	630 (21%)	2961

### WGD genes have more interaction partners on average than SSD genes

**Figure 3:**
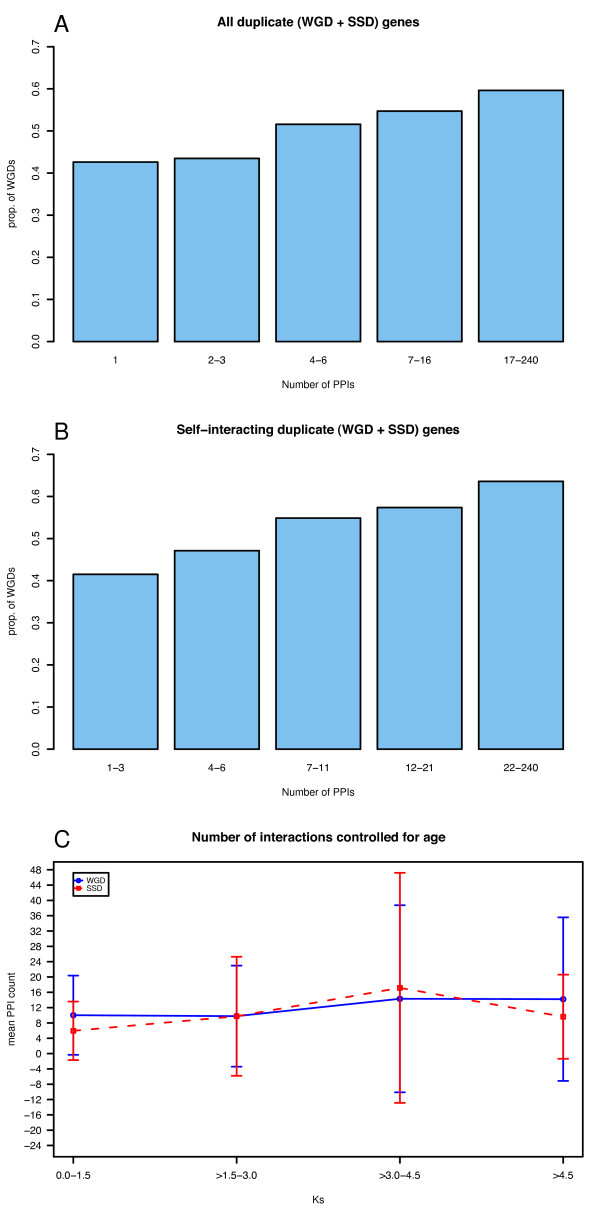
**Relationship of duplication type and number of interactions**. The proportion of WGD genes among all duplicate (WGD and SSD) genes increases with increased number of protein-protein interactions irrespective of self-interactions. a) Proportion of WGD genes among all duplicate genes with respect to the number of interactions. (Bins created to contain similar amounts of genes.) b) Proportion of self-interacting WGD genes among all self-interacting duplicate genes with respect to the number of interactions. (Bins created to contain similar amounts of genes.) c) Relationship of synonymous divergence rate, and the number of PPI partners of each gene in the duplicate pair. The x-axis displays the synonymous substitution rate (K_S_) between a duplicate pair, while the y-axis is the mean value of the total number of PPIs of all genes in each K_S _bin (category).

To further investigate the relationship of protein interaction network connectivity and duplicability we examined the number of interacting partners of duplicated genes. We find that the genes with a higher number of interactions contain a greater proportion of genes duplicated by WGD, and that this is true irrespective of self-interaction (p < 2 × 10^-16 ^and p = 1.03 × 10^-7 ^respectively, logistic regression; Figure 3a-b and Figure S2, Additional file 1). However, it was previously noted in yeast that older genes tend to have more interaction partners [21,22] and when we control for age of duplication, we find no difference in connectivity between all WGD and SSD duplicates (Figure 3c) or between self-interacting WGD and SSD duplicates (results not shown).

We therefore considered whether the enrichment for self-interactions is truly a generality of duplicated genes, or is only a feature of WGD-duplicated genes which are older on average than SSD-duplicated genes and tend to have a higher number of interaction partners. Indeed, the enrichment for self-interaction among WGD genes compared to singletons is highly significant (χ^2 ^= 55.88, df = 1, p = 7.69 × 10^-14^), but so too is the enrichment among SSD-duplicated genes (χ^2 ^= 19.92, df = 1, p = 8.08 × 10^-6^). Thus we conclude that self-interaction is a general feature of duplicated genes that is influenced by the mode of duplication and the total number of interactions, but not determined by those features.

### Self-interacting genes are enriched for developmental and essential biological processes, and WGD self-interacting genes are involved in metabolism

In order to understand the biological impact of duplication of self-interacting genes, we examined their functions using Gene Ontology (GO) terms. We found that self-interacting genes are enriched for biological processes such as early development (GO:0030154 and GO:0007275), cell death, cell communication and response to stimulus, and molecular functions such as protein binding, kinase and transferase activity as well as receptor activity (Table 3). We then compared duplicated against singleton genes, and found that while the first set of genes are over-represented for cell communication and cell differentiation, and molecular functions including binding, receptor activity and channel activity; they are under-represented for metabolic and catabolic processes, nucleic acid binding and ligase activity (Table 4). Finally we compared self-interacting WGD and SSD genes against each other, and found that while the WGD genes are under-represented for response to stimulus, oxidoreductase, anitoxidant and hydrolase activity the same set of genes are over-represented for regulation of biological processes, metabolic processes, kinase, transferase and transcription regulation activity (Table 5). Thus, genes involved in metabolic processes are under-represented among duplicate genes in general, but enriched in WGD-duplicated genes compared to SSD-duplicated genes. This is consistent with a recent report by Gout and colleagues which showed that after WGD, metabolic genes are retained more often than non-metabolic genes due to selection for gene expression on the entire metabolic pathway [7].

**Table 3:** Over-represented GO terms when self- and nonself-interacting genes are compared against each other.

*Biological Processes*						
GO IDs	Term	**Obs**.	Mean	**S. D**.	Z score	*p*-value^a^
GO:0008219	cell death	197	116.651	8.8	9.1	2.12E-16
GO:0007154	cell communication	588	480.984	15.4	6.9	2.78E-11
GO:0050789	regulation of biological process	855	748.686	15.3	7.0	4.33E-11
GO:0051704	multi-organism process	116	66.152	6.7	7.5	1.41E-10
GO:0006928	cell motion	113	71.586	7.0	5.9	8.45E-07
GO:0050896	response to stimulus	321	252.834	12.2	5.6	1.42E-06
GO:0007610	behavior	86	52.189	6.0	5.6	3.96E-06
GO:0009987	cellular process	833	764.383	15.3	4.5	8.67E-05
GO:0030154	cell differentiation	222	173.684	10.3	4.7	1.66E-04
GO:0043170	macromolecule metabolic process	697	628.169	15.9	4.3	1.79E-04
GO:0007275	multicellular organismal development	374	321.383	12.9	4.1	2.29E-03
GO:0032501	multicellular organismal process	222	188.816	10.8	3.1	4.20E-02

***Molecular Functions***						

**GO IDs**	**Term**	**Obs**.	**Mean**	**S. D**.	**Z score**	***p*-value^a^**

GO:0005515	protein binding	1026	874.792	14.8	10.2	1.25E-23
GO:0016301	kinase activity	208	118.109	9.3	9.7	2.34E-19
GO:0016740	transferase activity	213	145.362	10.3	6.6	1.52E-09
GO:0004871	signal transducer activity	113	75.003	7.3	5.2	2.24E-05
GO:0004872	receptor activity	208	169.553	11.0	3.5	1.22E-02

^a ^The estimated *P *values were adjusted by Bonferroni correction.

**Table 4:** Over- and under-represented (italics) GO terms when duplicated genes are compared against singleton genes.

*Biological processes*						
GO IDs	Term	**Obs**.	Mean	**S. D**.	Z score	*p*-value^a^
GO:0007154	cell communication	2009	1819.3	17.5	10.8	5.86E-27
*GO:0006139*	*nucleobase, nucleoside, nucleotide and nucleic acid metabolic process*	*1446*	*1630.6*	*16.6*	-*11.1*	*4.08E-25*
*GO:0009058*	*biosynthetic process*	*1522*	*1650.6*	*17.0*	-*7.6*	*2.21E-12*
GO:0050789	regulation of biological process	3130	3001.9	18.3	7.0	5.91E-11
GO:0007275	multicellular organismal development	1346	1241.1	15.8	6.7	1.76E-10
GO:0032501	multicellular organismal process	787	712.5	12.4	6.0	2.34E-08
*GO:0022904*	*respiratory electron transport chain*	*3*	*14.9*	*1.9*	-*6.2*	*1.45E-06*
*GO:0043170*	*macromolecule metabolic process*	*2628*	*2726.8*	*18.2*	-*5.4*	*1.72E-06*
GO:0030154	cell differentiation	726	673.2	12.1	4.4	3.02E-04
GO:0007610	behavior	218	189.6	7.1	4.0	3.39E-04
GO:0006928	cell motion	300	267.4	8.3	3.9	6.67E-04
*GO:0009056*	*catabolic process*	*452*	*487.6*	*11.0*	-*3.2*	*1.70E-02*
*GO:0005634*	*nucleus*	*1*	*5.1*	*1.2*	-*3.4*	*4.85E-02*
*GO:0016787*	*hydrolase activity*	*1*	*5.1*	*1.2*	-*3.4*	*4.85E-02*

***Molecular Function***						

**GO IDs**	**Term**	**Obs**.	**Mean**	**S. D**.	**Z score**	***p*-value^a^**

GO:0005488	binding	2905	2686.899	18.3	11.9	3.85E-29
GO:0004872	receptor activity	714	608.911	12.3	8.6	2.06E-19
GO:0016301	kinase activity	496	420.462	10.0	7.5	4.99E-14
GO:0015267	channel activity	203	159.644	6.6	6.6	1.28E-12
GO:0015075	ion transmembrane transporter activity	300	247.312	8.1	6.5	2.48E-11
GO:0004871	signal transducer activity	315	265.132	8.2	6.1	2.76E-09
*GO:0003676*	*nucleic acid binding*	*1211*	*1290.829*	*16.5*	-*4.8*	*3.31E-05*
GO:0016787	hydrolase activity	870	813.748	13.8	4.1	7.61E-04
*GO:0045182*	*translation regulator activity*	*36*	*51.717*	*3.6*	-*4.3*	*2.28E-03*
GO:0003774	motor activity	68	55.557	3.7	3.4	1.09E-02
*GO:0016874*	*ligase activity*	*138*	*161.46*	*6.4*	-*3.7*	*1.21E-02*

^a ^The estimated *P *values were adjusted by Bonferroni correction.

**Table 5:** Over- and under-representation (italics) of GO terms in self-interacting WGD with respect to SSD genes.

*Biological processes*						
**GO IDs**	**Term**	**Obs**.	**Mean**	**S. D**.	**Z score**	***p*-value^a^**

*GO:0050896*	*response to stimulus*	*138*	*171.285*	*7.5*	-*4.4*	*0.000246911*
GO:0050789	regulation of biological process	484	456.64	8.2	3.3	0.017011463
GO:0043170	macromolecule metabolic process	399	372.058	9.1	2.9	0.039105725

***Molecular Function***						

**GO IDs**	**Term**	**Obs**.	**Mean**	**S. D**.	**Z score**	***p*-value^a^**

GO:0016301	kinase activity	143	111.112	6.7	4.8	3.34E-05
GO:0016740	transferase activity	141	113.834	6.8	4.0	1.17E-03
GO:0030528	transcription regulator activity	134	107.982	6.7	3.9	1.67E-03
*GO:0016491*	*oxidoreductase activity*	*14*	*25.711*	*3.3*	-*3.6*	*1.93E-02*
*GO:0016209*	*antioxidant activity*	*0*	*4.858*	*1.5*	-*3.2*	*4.16E-02*
*GO:0016787*	*hydrolase activity*	*81*	*101.071*	*6.3*	-*3.2*	*4.37E-02*

^a ^The estimated *P *values were adjusted by Bonferroni correction.

## Conclusions

We observed greater duplicability of human self-interacting genes and that WGD duplicate genes tend to be self-interacting more often than SSD duplicate genes. This latter observation probably relates to the higher overall connectivity of WGD genes in protein interaction networks. Highly connected genes are more likely to be subject to dosage balance constraints and so to be resistant to SSD, but preferentially retained after WGD [4,8]. This result is also consistent with studies in yeast which showed preferential retention of interacting genes after WGD as a possible explanation of the protein interaction network dynamics [11]. Moreover, consistent with previous observations in yeast [21,22], we found that protein connectivity is correlated with the time since gene duplication. Our results also support the hypothesis that duplication of self-interacting proteins should be selectively advantageous because it facilitates the evolution of complex protein structures [2].

## Methods

### Filtering of human protein-protein interaction data

We obtained 37,107 PPIs involving 9303 genes from the Human Protein Reference Database (HPRD) release 7 [23,24]. We excluded interactions where either of the interacting partners could not be linked to an Ensembl release 50 identifier [25] as well as 13 genes that were too short or simple for the BLASTP sequence similarity search. Thus, the final dataset consisted of 34808 interactions, encoded by 8881 (1879 self-interacting) genes.

### Definition of singleton and duplicate genes

An all against all BLASTP search [26] of all known and novel human peptides present in Ensembl Core release 50 [25] was performed to define singleton and duplicate genes in human. Singleton genes were defined as genes whose protein products lack any non-self hit with an E-value less than 0.1. A gene was considered to be a duplicated gene if its top, non-self hit had an E-value less than or equal to 1 × 10^-20^, and at least 50% of the two peptides aligned. Genes with BLAST hits at intermediate E-values (less than 0.1 but larger than 1 × 10^-20^) were considered ambiguous genes, which could neither be classified as singleton or duplicate genes. The analyses were repeated with E-value thresholds of 1 × 10^-4^, 1 × 10^-10 ^and the results were consistent. Also, 102 genes lacked hits after the BLASTP search was performed (i.e., not even a self-hit). The reason for missing hits could all be assigned to low complexity (simple sequence) masking or too-short peptide sequences and these were excluded from further analysis.

### Definition of singleton and duplicate genes for the comparative study

Genes that have not recently duplicated in the human lineage (since the human-mouse split) were examined for mouse lineage-specific duplication using data from the Ensembl Compara release 50 [25] from human (*Homo sapiens*) and mouse (*Mus musculus*). A singleton gene was classified as a gene that had a one to one orthologous relationship between the two species, while a duplicate gene was a single human gene with at least two orthologous genes in mouse (1:many relationship; Figure 2).

### Comparison of WGD duplicate genes vs SSD duplicate genes

2877 WGD-duplicated genes (also known as ohnologs) with available PPI data were obtained from Nakatani et al. [27]. 307 genes that were not classified by us as duplicated based on our criteria of BLASTP sequence similarity and alignment length (above) were classified as WGD-duplicate genes based on Nakatani's analysis which includes comparative gene synteny. Thus the final dataset consisted of 2877 WGD genes and 2961 SSD-duplicated genes (Table 2).

We applied simple logistic regression [28,29] to measure the proportion of genes encoding self-interacting proteins over all genes with a particular number of interacting partners (degree) *k*, and in accordance with Ispolatov and colleagues [9] we found that self-interacting genes tend to have more interacting partners in general (not shown). We then measured the proportion of WGD genes over all duplicate genes (WGD and SSD genes) with degree *k*.

### Gene Ontology analyses

Gene Ontology (GO) terms related to biological process and molecular function were examined for sets of self- and nonself-interacting genes, self-interacting WGD and SSD genes, and duplicated and singleton genes using GO slim [30]converted by human GO identifiers [31] (both available from http://www.geneontology.org). Expected values were estimated by simulation, and p-values were calculated as the difference between the expected and observed under hypergeometric distribution. Finally the estimated p-values were adjusted by Bonferroni correction.

## Abbreviations

PPI: Protein-protein interaction; PIN: Protein interaction network; WGD: Whole genome duplication; SSD: Small scale duplication; GO: Gene ontology.

## Authors' contributions

ÅPB and AMcL devised the project. ÅPB and TM carried out experiments. ÅPB, TM and AMcL analysed the data. ÅPB and AMcL wrote the paper. All authors read and approved the final manuscript.

## Supplementary Material

Additional file 1 **Additional methods and results**. Click here for file
