# Supplementary material for: Duplicability of self-interacting human genes
Source: BMC Evol Biol. 2010 May 28;10:160. doi: 10.1186/1471-2148-10-160 (PMC2894830; doi:10.1186/1471-2148-10-160)
Supplement: Additional file 1 — Additional methods and results. [file 1471-2148-10-160-S1.DOC]

# Additional file 1

# Duplicability of self-interacting human genes

### Åsa Pérez-Bercoff, Takashi Makino and Aoife McLysaght

Smurfit Institute of Genetics, University of Dublin, Trinity College, Dublin 2, Ireland

Corresponding author: McLysaght, A. (aoife.mclysaght@tcd.ie)

# Supplementary Methods and Results

**Filtering of human protein-protein interaction data**

We used protein-protein interaction (PPI) data from the Human Protein Reference Database (HPRD) release 7 [1, 2]. Originally we had protein interaction information for 37107 interactions, involving 9303 genes (Figure S1). After discarding interactions where either gene lacked an Ensembl Core release 50 [3] identifier, the number of genes was reduced to 8894 (34900 interactions). Finally, 13 of those genes had no hit when a BLASTP search was performed, and since duplicate genes (see next section of methods) where defined using BLASTP search these 13 genes where removed. Thus, the final dataset consisted of 34808 interactions, encoded by 8881 (1879 self-interacting) genes.

**Definition of singleton and duplicate genes in human**

An all against all BLASTP search [4] of all known and novel human peptides present in Ensembl Core release 50 [3] was performed to define singleton and duplicate genes in human. Both the peptide and the gene it belonged to were extracted. Singleton genes were defined as genes lacking a hit when the E-value threshold was less than 0.1. For duplicate genes three different thresholds were introduced so that a gene was considered a duplicate if its top, non-self hit had an E-value less than or equal to 1x10^-4^, 1x10^-10^ and 1x10^-20^ respectively, and at least 50 % of the two peptides aligned. This generated three datasets (Table S1), each with the same threshold for defining singleton genes, but with different thresholds defining the duplicate genes. Genes with BLAST hits at intermediate E-values (less than 0.1 but larger than 1x10^-4^, 1x10^-10^ and 1x10^-20^ respectively) were considered ambiguous genes, which could neither be classified as singleton or duplicate genes. Genes that lacked hits after the BlastP search was performed were checked because if the search operated successfully we expected them to match themselves in the database, and the reason for missing hits could all be assigned to masking or too short peptide sequences.

Our analysis of the datasets showed that the association between self-interacting genes and duplicability holds true irrespectively of the E-value threshold chosen to define singleton and duplicate genes (χ^2^ = 45.24, df = 1, p = 1.74e-11; χ^2^ = 45.10, df = 1, p = 1.87e-11; and χ^2^ = 45.02, df = 1, p = 1.96e-11).

**Comparison of WGD duplicate genes vs SSD duplicate genes**

Frequency of protein self-interaction amongst human duplicate genes arisen through Whole Genome Duplication (WGD) also called *ohnologs* [5] and duplicates arisen through Smaller Scale Duplication (SSD) events were compared. Two separate paralogon datasets (Hokamp’s and Nakatani’s) were used to extract WGD genes. Only those genes that were classified as WGD genes in both paralogon datasets where considered reliable WGD genes.

In Hokamp’s dataset genes from Ensembl Core release 50 were used. *C. intestinalis* and Amphioxious, the latter from DOE Joint Genome Institute (http://www.jgi.doe.gov/), served as outgroups, and the method for detecting WGD genes was modified from [6]. Only paralogons consisting of at least 6 distinct duplicated genes were used. We found that from our previous definitions (Table S1) we had to reclassify 249 (49 self-interacting) of the 2595 singleton genes, 89 (18 self-interacting) of the 755 ambiguous genes and 2039 (516 self-interacting) of the 5531 duplicate genes (Table S1, set 3). Thus the final dataset consisted of 2377 WGD genes (583 self-interacting) and the original 5531 duplicate genes the remaining 3492 (769 self-interacting) where defined as SSD genes (Table S2).

The second WGD data set, obtained from Yoichiro Nakatani, and derived from his method [7], consisted of 4901 WGD genes. After matching these genes against HPRD release 7 and Ensembl release 50 identifiers 2877 (717 self-interacting) WGD genes remained in the set. Of these 274 (53 self-interacting) had previously been classified as singleton genes, 33 (9 self-interacting) as ambiguous genes and 2570 (655 self-interacting) as duplicate genes. Thus of the original 5531 duplicate genes 2961 (630 self-interacting) where defined as SSD genes (Table S2).

1511 genes (402 self-interacting) were classified as WGD genes by both Hokamp’s and Nakatani’s methods, and hence we considered them as reliable WGD genes. Of these 1511 genes, 1357 had previously been classified as belonging to the 5531 duplicate genes, and thus as the remaining 4174 duplicate genes (918 self-interacting) were not defined as WGD genes by either method they were defined as SSD genes.

**Statistical comparisons of interactions of duplicated and singleton genes and of WGD and SSD genes**

To calculate whether the observed number of duplicate genes with self-interactions were what we would expect to observe due to chance variation we performed Pearson’s χ^2^-test with 1 degree of freedom (df), and a significance level of α=0.05. This statistical test was performed on our three human datasets, on our comparative human-mouse dataset, and finally on our three ohnolog datasets where WGD duplicate genes were compared to SSD duplicate genes. We compared singleton and duplicate genes, genes encoding for self-interacting proteins and genes lacking self-interacting proteins. We found a strong association indicating that WGD duplicate genes tend to be self-interacting more often than SSD duplicate genes (χ^2^ = 4.87, df = 1, p = 0.027; (χ^2^ = 10.67, df = 1, p = 0.0011; (χ^2^ = 12.98, df = 1, p = 0.00032).

**Controlling for age (synonymous substitution rate) and protein connectivity**

From the previously conducted all-against-all BLASTP search we extracted the best non-self hit with at least 50 % alignment as the closest paralog of each gene in order to create duplicate gene pairs. Afterwards the genes of each pair were classified according to our previous gene type classification (singleton, ambiguous, duplicate, WGD or SSD). Since we wanted to compare the connectivity of WGD and SSD genes of similar age we calculated the synonymous substitution rate (K_S_) of gene pairs where both genes where either WGD or SSD genes. The synonymous substitution rate (K_S_) was estimated by the Yang & Nielsen method [8], which is implemented in PAML [9]. Pairs with (K_S_) values of -0 or 99 (saturated) were excluded. Thus two datasets of WGD (2139 pairs) and SSD (1519 pairs) duplicate pairs were created, and a histogram was drawn for each dataset (Figure S3). Subsequently a line plot with two series (WGD and SSD duplicate pairs respectively) was drawn where the synonymous substitution rate (K_S_) of each duplicate pair was plotted against the mean value of the total PPI number of each (K_S_) category (Figure 3c).

As been previously shown in yeast [10, 11] we found that in human there is also a correlation between age (divergence rate (K_S_) of a gene) and the connectivity level of the protein the gene is encoding although the trend (Figure 3c) is unclear. Furthermore there is a bias of duplication timing for WGD genes i.e. WGD genes tends to be older than SSD genes. In particular, there are few WGD genes with K_S_ <1.5, which have low connectivity. These results indicate that K_S_ bias cause a difference in the total connectivity between WGD and SSD genes respectively.

**References**

1. Keshava Prasad TS, Goel R, Kandasamy K, Keerthikumar S, Kumar S, Mathivanan S, Telikicherla D, Raju R, Shafreen B, Venugopal A *et al*: Human Protein Reference Database--2009 update. *Nucleic Acids Res* 2009, 37(Database issue):D767-772.

2. Peri S, Navarro JD, Amanchy R, Kristiansen TZ, Jonnalagadda CK, Surendranath V, Niranjan V, Muthusamy B, Gandhi TK, Gronborg M *et al*: Development of human protein reference database as an initial platform for approaching systems biology in humans. *Genome Res* 2003, 13(10):2363-2371.

3. Flicek P, Aken BL, Beal K, Ballester B, Caccamo M, Chen Y, Clarke L, Coates G, Cunningham F, Cutts T *et al*: Ensembl 2008. *Nucleic Acids Res* 2008, 36(Database issue):D707-714.

4. Altschul SF, Gish W, Miller W, Myers EW, Lipman DJ: Basic local alignment search tool. *J Mol Biol* 1990, 215(3):403-410.

5. Wolfe KH: Robustness - it's not where you think it is. *Nature Genetics* 2000, 25.

6. McLysaght A, Hokamp K, Wolfe KH: Extensive genomic duplication during early chordate evolution. *Nat Genet* 2002, 31(2):200-204.

7. Nakatani Y, Takeda H, Kohara Y, Morishita S: Reconstruction of the vertebrate ancestral genome reveals dynamic genome reorganization in early vertebrates. *Genome Res* 2007, 17(9):1254-1265.

8. Yang Z, Nielsen R: Estimating synonymous and nonsynonymous substitution rates under realistic evolutionary models. *Mol Biol Evol* 2000, 17(1):32-43.

9. Yang Z: PAML: a program package for phylogenetic analysis by maximum likelihood. *Comput Appl Biosci* 1997, 13(5):555-556.

10. Kunin V, Pereira-Leal JB, Ouzounis CA: Functional evolution of the yeast protein interaction network. *Mol Biol Evol* 2004, 21(7):1171-1176.

11. Prachumwat A, Li WH: Protein function, connectivity, and duplicability in yeast. *Mol Biol Evol* 2006, 23(1):30-39.

Figures

## Figure S1 - Detailed flow chart illustrating collection and filtering of genes with protein-protein interaction information

Flow chart illustrating how the human interaction data was collected from HPRD release 7, and all the subsequent filtering steps when the genes where matched to blastable Ensembl Core release 50 identifiers.


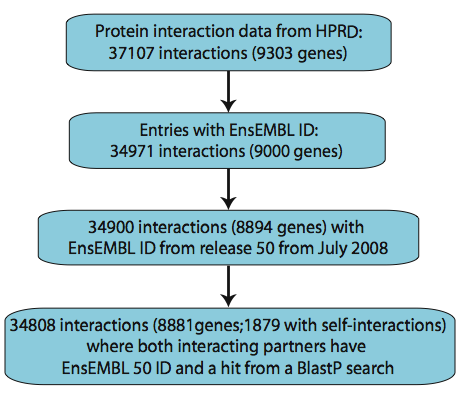


## Figure S2 - Increased proportion of WGD genes with increased protein connectivity

Protein connectivity (ln(PPIs)) plotted against the proportion of WGD genes (logistic regression).

1. The proportion of self-interacting WGD genes over all self-interacting duplicate (WGD and SSD) genes increases with the number of PPIs of the genes.
2. The proportion of nonself-interacting WGD genes over all nonself-interacting (WGD and SSD) genes increases with the number of PPIs of the genes.
3. The proportion of WGD genes over all duplicate (WGD and SSD) genes increases with the number of PPIs of the genes.


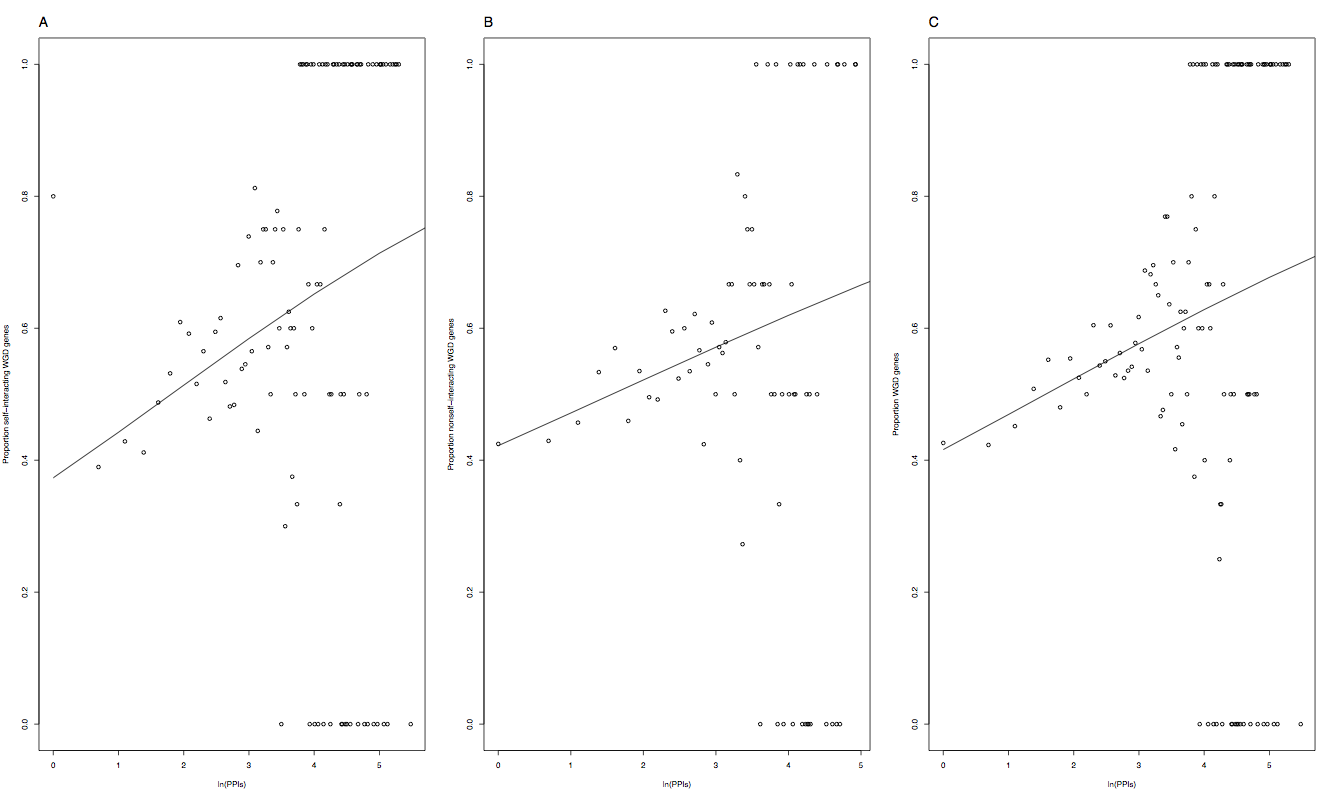


**Figure S3 – Synonymous divergence distribution for WGD and SSD duplicate pairs.**

The percentage of (A) WGD and (B) SSD duplicate genes with differing degrees of synonymous divergence.The x-axis indicates the synonymous substitution rate (K_S_) between a duplicate gene pair, and the y-axis percentage of duplicates with that K_S_.


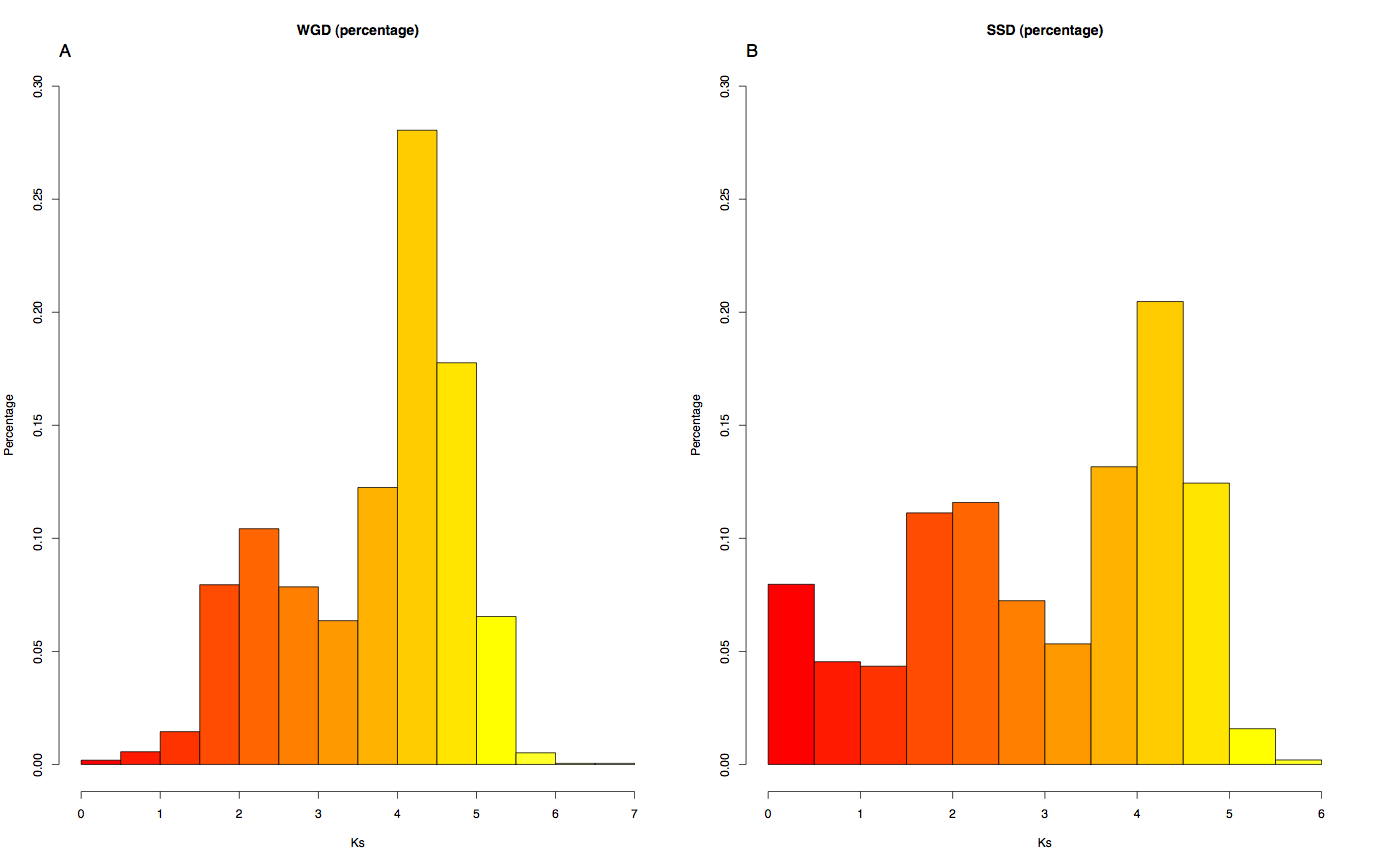


**Tables**

Table S1 – Frequency of self-interaction of singleton and duplicate genes under alternative parameters for paralog definition

| Set | E-value | singletons | ambiguous | duplicates | genes/set | Tot # genes |
| --- | --- | --- | --- | --- | --- | --- |
|  |  | self/nonself/tot |  | self/nonself/tot |  |  |
| 1 | 1x10^-4^ | 433/2162/2595 | 104 | 1431/4751/6182 | 8777 | 8881 |
| 2 | 1x10^-10^ | 433/2162/2595 | 375 | 1370/4541/5911 | 8506 | 8881 |
| 3 | 1x10^-20^ | 433/2162/2595 | 755 | 1285/4246/5531 | 8126 | 8881 |

Table S2 – Frequency of self-interactions of WGD and SSD duplicate genes under alternative definitions of WGD genes

| Set | WGD dupl | SSD dupl | Tot # genes |
| --- | --- | --- | --- |
|  | self/nonself/tot | self/nonself/tot |  |
| Nakatani's | 717/2160/2877 | 630/2331/2961 | 5838 |
| Hokamp's | 583/1794/2377 | 769/2723/3492 | 5869 |
| combined | 402/1109/1511 | 481/1798/2279 | 3790 |
